# Norcantharidin Enhances the Antitumor Effect of 5-Fluorouracil by Inducing Apoptosis of Cervical Cancer Cells: Network Pharmacology, Molecular Docking, and Experimental Validation

**DOI:** 10.3390/cimb46050242

**Published:** 2024-04-25

**Authors:** Yong Huang, Xin-Wei Wan, Yu-Tong Du, Yue Feng, Lin-Sen Yang, Yong-Bin Liu, Tian Chen, Zhuan Zhu, Yi-Ting Xu, Cheng-Cheng Wang

**Affiliations:** GuiZhou University Medical College, Guiyang 550025, China; hy930041043@163.com (Y.H.); wgswxw@126.com (X.-W.W.); dyt630876889@163.com (Y.-T.D.); fengyue0224@163.com (Y.F.); 18585614832@163.com (L.-S.Y.); liuyb1020@163.com (Y.-B.L.); 18925832731@163.com (T.C.); 18386569021@163.com (Z.Z.); xuyiting0321@163.com (Y.-T.X.)

**Keywords:** nature product, synergism, cancer, HPV-positive, network pharmacology, bioactivity

## Abstract

The high recurrence rate of cervical cancer is a leading cause of cancer deaths in women. 5-Fluorouracil (5-FU) is an antitumor drug used to treat many types of cancer, but its diminishing effectiveness and side effects limit its use. Norcantharidin (NCTD), a demethylated derivative of cantharidin, exhibits various biological activities. Here, we investigated whether NCTD could potentiate 5-FU to induce cervical cancer cell death. To assess the cell viability and synergistic effects of the drugs, cell counting kit-8 and colony formation assays were performed using HR-HPV-positive cervical cancer cell lines. Annexin V-FITC/PI staining and TUNEL assays were performed to confirm the induction of apoptosis. The synergistic effect of NCTD on the antitumor activity of 5-FU was analyzed using network pharmacology, molecular docking, and molecular dynamics simulations. Apoptosis-related proteins were examined using immunoblotting. The combination of NCTD and 5-FU was synergistic in cervical cancer cell lines. Network pharmacological analysis identified 10 common targets of NCTD and 5-FU for cervical cancer treatment. Molecular docking showed the strong binding affinity of both compounds with CA12, CASP9, and PTGS1. Molecular dynamics simulations showed that the complex system of both drugs with caspase-9 could be in a stable state. NCTD enhanced 5-FU-mediated cytotoxicity by activating apoptosis-related proteins. NCTD acts synergistically with 5-FU to inhibit cervical cancer cell proliferation. NCTD enhances 5-FU-induced apoptosis in cervical cancer cell lines via the caspase-dependent pathway.

## 1. Introduction

Cervical cancer is one of the most common malignancies in women worldwide [1]. Persistent infection with high-risk human papillomavirus (HR-HPV) is associated with cervical cancer [2]. Despite advances in HPV vaccine research and application [3], not all HR-HPV types are covered, and these vaccines do not prevent the progression to cervical cancer in individuals who are already infected [4]. Cervical cancer mortality rates have not changed significantly in recent decades [5].

Chemotherapy and radiotherapy are the standard treatments for most patients with cervical cancer [6]. Chemotherapy plays a central role in the treatment of advanced and metastatic cervical cancer [7]. First-line chemotherapy agents are usually the first choice, but the use of a second-line agent can be justified in certain circumstances, such as when a patient is insensitive or resistant to first-line treatment. 5-Fluorouracil (5-FU), an analogue of uracil, has been widely used alone or in combination with other therapeutic agents for the clinical treatment of several types of cancer, including colorectal cancer [8], gastric cancer [9], liver cancer [10], and cervical cancer [11]. The primary mechanisms underlying anticancer activity involve inhibiting thymidylate synthase activity and directly incorporating its metabolites into the DNA and RNA of cancer cells, thereby impairing DNA synthesis and repair [12]. However, 5-FU-based chemotherapy has limited anticancer efficacy in clinical conditions due to resistance and dose-limiting cytotoxicity [12]. An optimal approach to address these challenges is to combine 5-FU with other anticancer drugs that exhibit distinct mechanisms of action. This strategy aims to overcome the limitations of 5-FU monotherapy.

Mylabris is the dried body of the Chinese blister beetle, which is one of the earliest antitumor medicines discovered in China. The species of Mylabris used in medicine are usually *Mylabris phalerata* Pallas and *Mylabris cichorii* Linnaeus. Cantharidin (CTD), one of the main anticancer active ingredients, is extracted from them [13,14]. However, its clinical application is limited by toxicity issues and synthesis complexity. In contrast, NCTD has the advantages of easy synthesis, fewer side effects, and stronger activity than cantharidin [15]. Specially, the Diels–Alder reaction between furan and maleic anhydride resulted in 5,6-dehydro-norcantharidin, which was subsequently reduced to yield NCTD [16]. In addition, norcantharidin has nearly the same anticancer activity as cantharidin and relatively less toxicity and fewer side effects and has been successfully utilized as an effective anticancer drug in clinical practice [14,15,17]. However, the effect of NCTD on human cervical cancer cells remains largely unknown.

NCTD affects the expression of caspase-9 and caspase-3, which induces apoptosis [18]. In mammals, two apoptotic pathways have been well documented: the extrinsic pathway, which is initiated by receptors, and the intrinsic pathway, which is triggered by mitochondrial stress. Both pathways employ a cascade of executioner proteases, commonly called caspases, to accomplish the ultimate breakdown of cellular structures. Upon the activation of the signal transduction pathway, caspases are activated, triggering a cascade of apoptotic proteases that irreversibly drive cells into apoptosis [19]. Caspase-9, a crucial caspase initiator involved in the initiation of apoptosis via the mitochondrial pathway, is activated upon association with the apoptosome complex. Caspase-9 is required in most scenarios of apoptotic cell death, and consequently, its activation has profound consequences [20]. Caspase-9 can activate downstream caspase-3 proteases, which eventually induce apoptosis [19,20,21]. However, the potential molecular mechanisms of action of NCTD in combination with 5-FU in cervical cancer cells remain to be elucidated.

Network pharmacology is a new approach to predict the potential targets and active components of traditional Chinese medicines (TCMs), which can facilitate the exploration of their molecular mechanisms [22]. Molecular docking is commonly used to explore the affinity binding sites between the active ingredients of natural products and disease targets [23]. Several studies have demonstrated the effects of natural products on tumor cell death using network pharmacology. Molecular dynamics simulations can identify the trajectory of and temporal changes in the complex, as well as discover atomic interactions between ligands and protein amino acid residues, which can validate and complement molecular docking results. For instance, Xu et al. found that *Salvia miltiorrhiza* Bunge could inhibit ovarian cancer via the PI3K-Akt signaling pathway [24]. Using network pharmacology, Hu et al. revealed that wolfberries can treat breast cancer by acting on ESR1, MYC, and other targets [25].

In the present study, we revealed how NCTD and 5-FU, in combination, exhibited a strong synergistic effect on cervical cancer cells. Network pharmacology, molecular docking, and molecular dynamics simulation were employed to explore the core targets and mechanisms of 5-FU in combination with NCTD against cervical cancer. Furthermore, experimental validation demonstrated that NCTD enhances the anticancer activity of 5-FU by promoting apoptosis through the caspase-dependent pathway.

## 2. Materials and Methods

### 2.1. Reagents and Antibodies

Stock solutions of NCTD (Yuanye Bio-Technology Co., Ltd., Shanghai, China), 5-FU (Selleck Biotech Co., Ltd., Houston, TX, USA), and DMSO (Sigma-Aldrich, St. Louis, MO, USA) were prepared. The preparation of NCTD and 5-FU stock solutions: 20 mg NCTD powder was diluted to 100 mM with 1.2 mL DMSO. The working concentration was 12.5/25 μM. Analogously, 20 mg 5-FU powder was diluted to 100 mM with 1.5 mL DMSO. The working concentration of 5/10 μM was then prepared. Dulbecco’s modified Eagle’s medium (DMEM), fetal bovine serum (FBS), penicillin/streptomycin (PS), and trypsin were obtained from Gibco, Thermo Fisher Scientific, Inc. (Waltham, MA, USA). β-actin monoclonal antibodies were purchased from Proteintech Group, Inc. (Rosemont, IL, USA). Antibodies against caspase-3 and caspase-9 were purchased from Cell Signaling Technology Inc. (Boston, MA, USA). Goat anti-rabbit IgG-HRP and goat anti-mouse IgG-HRP were purchased from Jackson ImmunoResearch Inc. (West Grove, PA, USA). Cell counting kit-8 (CCK-8) was purchased from Beyotime Biotechnology (Shanghai, China). The Annexin V-FITC/PI Apoptosis Detection Kit and TUNEL FITC Apoptosis Detection Kit were purchased from Vazyme Biotech Co., Ltd. (Nanjing, China).

### 2.2. Cell Lines and Cell Culture

HeLa and SiHa human cervical cancer cell lines were purchased from the Chinese Academy of Sciences Cell Bank (Shanghai, China). Cells were cultured in DMEM supplemented with 10% FBS and 1% PS in 5% CO_2_ at 37 °C.

### 2.3. Cell Viability Assay

Cell counting kit-8 (CCK-8) assay was performed to investigate the cytotoxicity of NCTD, 5-FU, and their combination on HeLa and SiHa cells. Cells (4000/well) were seeded in 96-well plates and incubated for 24 h. Then, the drug-containing medium was administrated to the cells and incubated for another 24, 48, and 72 h at 37 °C. Subsequently, CCK-8 solution was added to the cells, which were then incubated for 1 h. The optical density (OD) was measured at 450 nm using a microplate reader (Bio-Rad Laboratories, Hercules, CA, USA). The equation used to calculate cell viability was as follows: cell viability (%) = (OD (treated) − OD (blank))/(OD (control) − OD (blank)) × 100%, where “blank” indicates PBS only, “treated” indicates drug-containing medium, and “control” indicates medium. The cell counting kit-8 (CCK-8) assay was performed to investigate the cytotoxicity.

### 2.4. Combination Index (CI)

The combination index (CI) of NCTD and 5-FU was calculated using CompuSyn software 1.0. A CI < 1 indicated a synergistic effect, whereas a CI = 1 or >1 indicated additive or antagonistic effects.

### 2.5. Colony Formation Assay

HeLa and SiHa cells were seeded in 6-well plates at a density of 1000 cells/well. After overnight incubation, the cells were treated with 5-FU, NCTD, or their combination for 72 h. The solutions containing drugs were replaced with fresh medium, and cells were cultured for two weeks, with the medium being changed every three days. Cell colonies were fixed in 4% paraformaldehyde for 20 min and stained with 0.1% crystal violet for 15 min. Finally, a high-resolution digital camera was used to capture the images.

### 2.6. Annexin V-FITC/PI Staining Assay

An Annexin V-FITC/PI staining assay was performed to detect apoptosis in HeLa and SiHa cells. Cells (1 × 10^5^/well) were seeded in a 12-well plate and incubated overnight. The drug-containing medium was then added to the cells and incubated for another 72 h at 37 °C. Cells were then harvested and stained according to the manufacturer’s instructions. Detection was performed using a BD JAZZ flow cytometer (Becton, Franklin Lakes, NJ, USA).

### 2.7. Terminal Deoxynucleotidyl Transferase dUTP Nick end Labeling (TUNEL) Assay

Cells were seeded onto confocal Petri dishes. After 24 h, medium-containing drugs (NCTD, 5-FU, and their combination) were added to the cells and co-incubated for another 72 h. The TUNEL assay was performed according to the manufacturer’s instructions. Images were captured with a confocal laser scanning microscope (Olympus Corp., Tokyo, Japan).

### 2.8. Network Pharmacology Analysis

The pharmacological targets of NCTD and 5-FU were screened using TargetNet [26]. Genes associated with apoptosis were extracted from the Genecard database [27]. Subsequently, a comparison was conducted to identify the overlapping targets between the drug targets and apoptosis-related genes. The common targets of the NCTD and 5-FU treatments and apoptosis were explored using the STRING database to construct a target-related protein interaction network and a protein–protein interaction (PPI) network map and .tsv data [28]. The topological parameters were analyzed using the network analyzer setting in Cytoscape [29], and core targets were selected based on the MCC algorithm.

### 2.9. Molecular Docking

Molecular docking was used to analyze the binding modes of NCTD and 5-FU to the targets. The protein structures were obtained from the Protein Data Bank (PDB) database [30] (BCL2A1:6VO4, CA12:5LL5, CASP9:2AR9, CES1:5A7G, CYP19A1:3S79, CYP1A2:2HI4, PTGS1:6Y3C, and SIRT2:5Y5N). PyMOL software 2.2.0 was used to eliminate water molecules and small molecule ligands from the protein structures. The AutoDockTools software 1.5.6 was used to add hydrogen ions and determine the location of the target protein activity pockets. Molecular docking was performed using the AutoDockVina software [31,32]. The docking models were visualized using PyMOL and Discovery Studio Visualiser 4.5. TBtools [33] software 2.042 was used to generate heat maps of the binding energies.

### 2.10. Molecular Dynamics Simulations

We used the GROMACS 2022.4 software to perform all-atom molecular dynamics simulations of protein–ligand complexes [34]. The protein part was parameterised using the Amber14SB force field, while the topology files for the small molecule drugs (NCTD or 5-FU) were generated using ACPYPE with the Antechamber program [35]. We chose a cubic solvation box and set the minimum 1 nm distance between the edge of the system to the complex. We selected the TIP3P-dominating water model and added appropriate amounts of sodium and chloride ions to balance the charge of the simulated system. Furthermore, the system was energy minimised using the steepest descent method. We maintained the temperature and pressure at 300 K and 101.325 kPa under constant temperature (NVT) and constant pressure (NPT). For each equilibrated system, we performed 10 ns molecular dynamics simulations at 300 K, obtaining a total of 1000 frames of simulated trajectories. Using the trajectory data obtained from the simulations, we performed an in-depth analysis of key parameters such as root mean square deviation (RMSD), root mean square fluctuation (RMSF), the radius of gyration (Rg), and the number of hydrogen bonds between proteins and small molecule drugs (NCTD or 5-FU).

### 2.11. Immunoblotting Analysis

Protein samples were separated by sodium dodecyl sulfate–polyacrylamide gel electrophoresis (SDS-PAGE) performed on a polyvinylidene fluoride membrane. The membranes were blocked with 5% skim milk in TBST buffer for 1 h. The membrane was then incubated with different primary antibodies for 2 h, followed by incubation with secondary antibodies for 1 h. Proteins were detected using an enhanced chemiluminescence system (Tanon, Shanghai, China).

### 2.12. Statistical Analysis

One-way and two-way analyses of variance (ANOVA) were performed using GraphPad Prism software, version 8 (GraphPad Software, San Diego, CA, USA). All experiments were performed in triplicate. Data are presented as the mean, with error bars representing the standard error of the mean (SEM) calculated from at least three independent experiments. The statistical significance was set at *p* < 0.05 significant.

## 3. Results

### 3.1. The Single-Drug Treatment of NCTD or 5-FU Increases the Cytotoxic of Cervical Cancer Cell Lines

**Figure 1:**
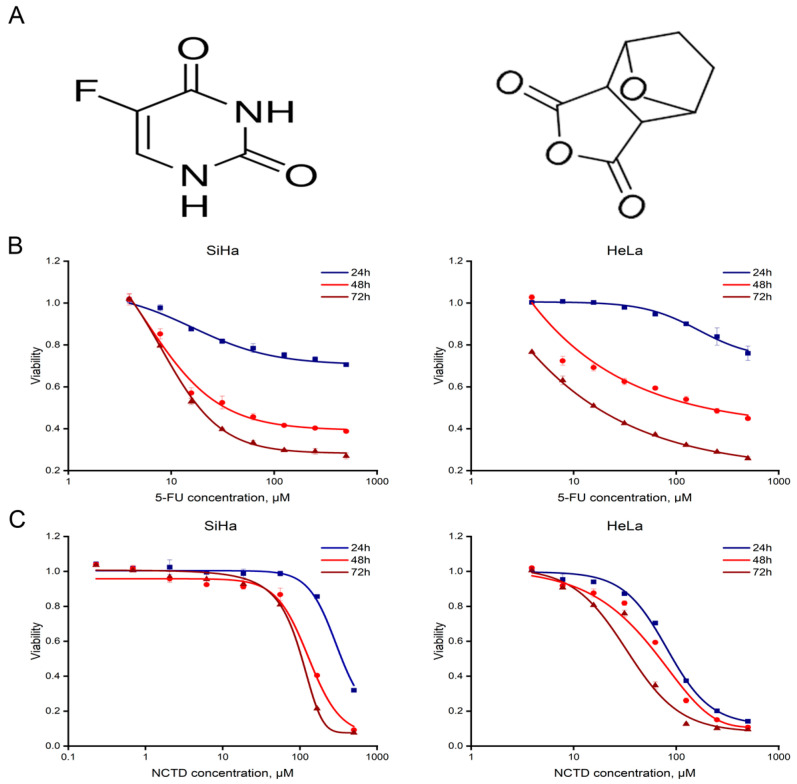
5-FU and NCTD inhibit cervical cancer cell proliferation. (**A**) The structures of 5-FU (**left**) and NCTD (**right**). (**B**) Cell viability was measured by the CCK-8 assay after 5-FU treatment of SiHa and HeLa cells. (**C**) Cell viability was measured by the CCK-8 assay after NCTD treatment of SiHa and HeLa cells.

To determine a sufficient and appropriate dose of 5-FU (Figure 1A, left) and NCTD (Figure 1A, right) in combination for cell viability, HeLa cells were treated with NCTD and 5-FU at different concentrations (0.82–500 μM, 3.91–500 μM respectively) for 24, 48, and 72 h. Similarly, SiHa cells were treated with NCTD and 5-FU at different concentrations (0.23–500 μM) for 24, 48, and 72 h (Figure 1B,C). After incubation, cell viability was assessed using the CCK-8 assay. The results indicate that both NCTD and 5-FU significantly inhibited cell proliferation in a time- and dose-dependent manner and significantly decreased cell viability. Exposure to 72 h treatments of 5-FU and NCTD exhibited greater antiproliferative effects, as evidenced by the IC50 of the drugs. The IC_50_ value of NCTD was 40.96 ± 5.33 μM in HeLa cells and 100.12 ± 0.30 μM in SiHa cells. The IC_50_ values of 5-FU in HeLa and SiHa cells were 5.96 ± 0.33 μM and 4.52 ± 0.30 μM, respectively. However, the IC_50_ values for the 24 and 48 h treatments were much higher.

These results provide a basis for the subsequent selection of drug dosages in combination. In conclusion, these findings provide evidence that each drug individually decreases the viability of cervical cancer cells.

### 3.2. The Combination of NCTD and 5-FU Is Synergistic in Cervical Cancer Cell Lines

**Figure 2:**
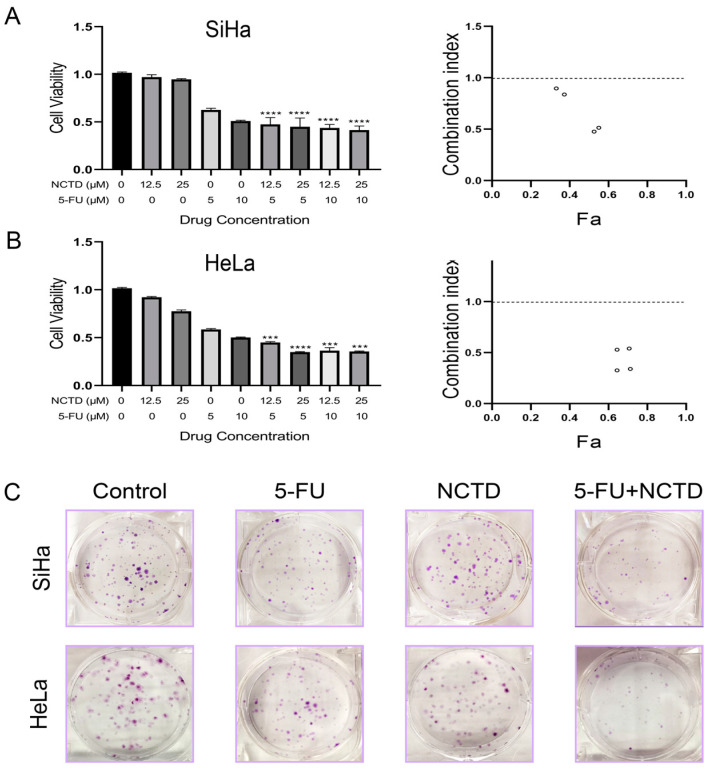
The synergistic effects of NCTD, 5-FU, or in combination on the cytotoxicity of HeLa and SiHa cells. (**A**,**B**) SiHa ((**A**) **left**) and HeLa ((**B**) **left**) cells were cultured for 72 h with NCTD (12.5 and 25 μM) or 5-FU (5 and 10 μM) alone and in combination. Cell survival was measured by the CCK-8 assay. Date are shown as the mean ± SEM (n = 3): *** *p* < 0.001, and **** *p* < 0.0001 versus 5-FU group. Dot plot summary showing the CI values of NCTD and 5-FU on SiHa ((**A**) **right**) and HeLa ((**B**) **right**). The CI values were analyzed using CompuSyn software, and the CI was interpreted as follows: synergy (<1), addition (=1), and antagonism (>1). (**C**) Colony formation of SiHa and HeLa cells after NCTD, 5-FU, or the combination treatment.

To evaluate the synergistic effects of NCTD and 5-FU on cervical cancer cells, a CCK-8 assay was performed using HeLa and SiHa cells. We selected two concentrations of NCTD (12.5 and 25 μM) and two concentrations of 5-FU (5 and 10 μM) to evaluate the effects of the combined drugs at each of these concentrations relative to those of the individual drugs. The results showed decreased cell viability with the combination treatment compared to NCTD or 5-FU monotherapy (Figure 2A,B left). To quantify the potential synergistic interaction of the NCTD + 5-FU combination, CompuSyn software was used to analyze the CI value. NCTD showed a synergistic cytotoxic effect in combination with 5-FU in all tested cell lines, as evidenced by a CI value of less than 1 (Figure 2A,B right).

The colony formation assay was performed to confirm the effect of the combination of NCTD and 5-FU on the survival of cervical cancer cells. As expected, the combination treatment significantly reduced the clonogenic survival of HeLa and SiHa cells (Figure 2C). Taken together, these results suggest that the combination of NCTD and 5-FU has antiproliferative activity in cervical cancer cells.

### 3.3. NCTD Increased 5-FU-Mediated Cytotoxicity through the Induction of Apoptosis

**Figure 3:**
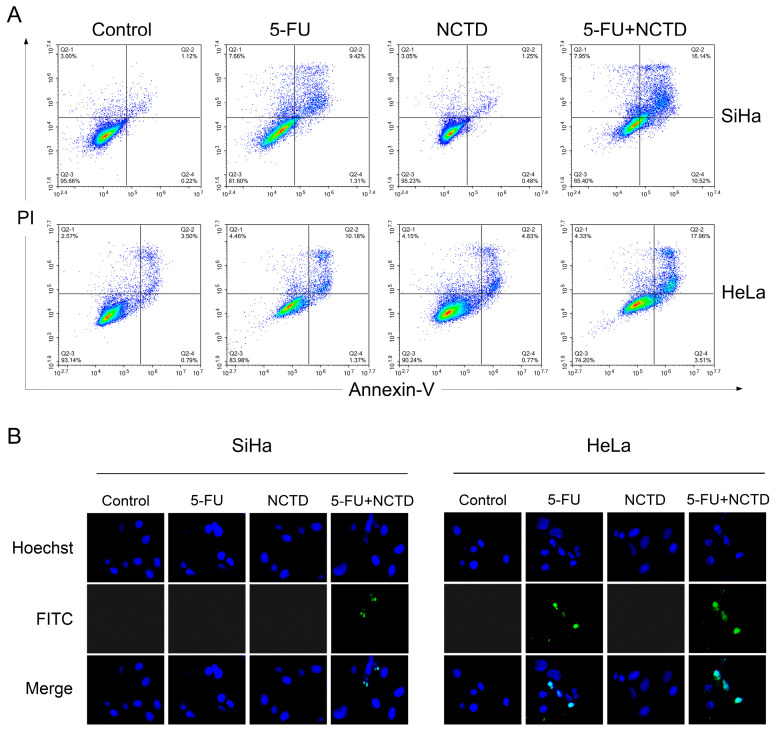
The combination of NCTD with 5-FU synergistically increased the proportion of cell apoptosis in HeLa and SiHa cells. (**A**) Flow cytometric analysis of Annexin V-FITC/PI-stained HeLa and SiHa cells after 72 h of NCTD, 5-FU, or the combination treatment. (**B**) TUNEL analysis of HeLa and SiHa cells treated with NCTD, 5-FU, or the combination treatment. FITC-stained cells are green, and Hoechst-stained nuclei are in blue (Magnification, ×20).

An Annexin V-FITC/PI staining assay was performed to confirm the induction of apoptosis by NCTD + 5-FU. HeLa and SiHa cells were incubated with NCTD, 5-FU, or a combination of NCTD and 5-FU. We observed a significant increase in apoptotic cervical cancer cells after 72 h of the NCTD + 5-FU treatment (Figure 3A). This synergistic effect was also demonstrated using the TUNEL assay. As shown (Figure 3B), the combination of NCTD and 5-FU resulted in an increase in FITC-positive cells compared to the treatment groups alone, suggesting an enhanced induction of apoptosis in the combination group.

### 3.4. Network Pharmacological Analysis Showed That the Combination of NCTD and 5-FU May Induce Apoptosis of Cervical Cancer Cells by Regulating the Caspase-Dependent Pathway

**Figure 4:**
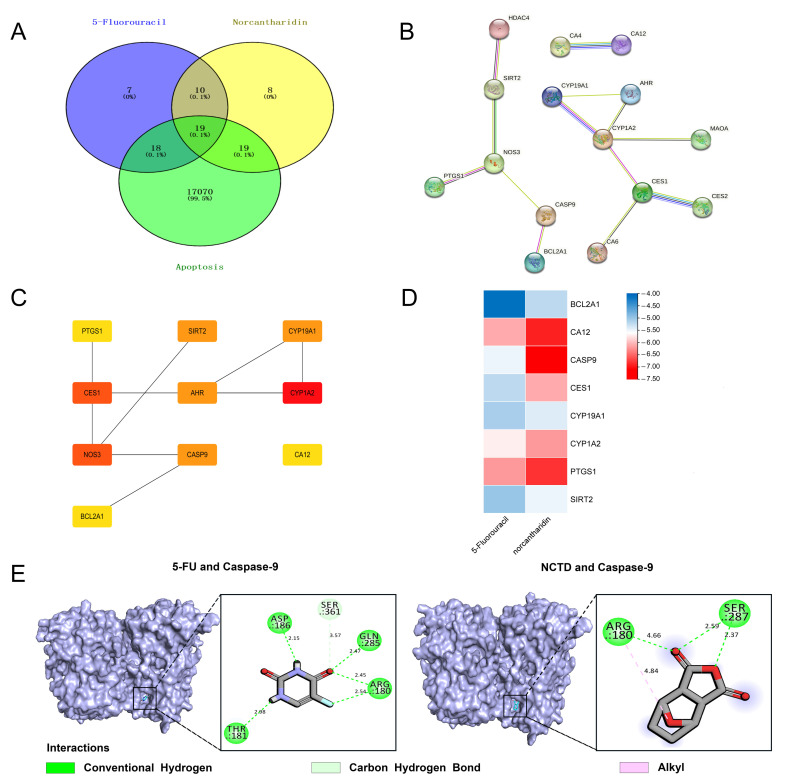
Network pharmacology analysis of NCTD- and 5-FU-induced apoptosis in HeLa and SiHa cells. (**A**) The resulting graph of the intersection between the potential targets of A and B and apoptosis-related genes. (**B**) PPI network of 19 overlapping genes. (**C**) Identification of core genes from the 19 selected genes. (**D**) Molecular docking analysis of NCTD and 5-FU with 10 core proteins. (**E**) Binding models of NCTD and 5-FU with caspase-9, respectively.

We conducted a network pharmacology analysis to gain insight into the targeting mechanisms of NCTD and 5-FU in inducing apoptosis. Initially, we collected data on 17,126 genes associated with apoptosis from GeneCards. TargetNet database was used to determine the pharmacological targets of NCTD and 5-FU. By comparing these three gene clusters, 19 intersecting genes were identified between the drug targets and apoptosis (Figure 4A). Subsequently, STRING analysis was employed to investigate the PPI network mediated by the 19 intersection targets involved in NCTD- and 5-FU-induced cell apoptosis (Figure 4B). All mapped intersecting proteins were input into Cytoscape software 3.6.1 to calculate the topological parameters of the PPI network related to NCTD- and 5-FU-induced cell apoptosis. The analysis identified ten hub gene targets (Figure 4C). Molecule docking analysis assessed the potential binding of NCTD and 5-FU to the identified hub targets and their involvement in cell apoptosis, except NOS3 and AHR, for which a software error was encountered. The results demonstrated that NCTD exhibited strong binding affinity to CA12, CASP9, and PTGS1 (Figure 4D). Caspase-9 plays a crucial role in the induction of the apoptosis process [36]. Therefore, we visualized the binding of these two drugs to caspase-9. The hydrogen bonds formed between 5-FU and caspase-9 occurred between the THR-181 (2.98 Å), ASP-186 (2.15 Å), GLN-285 (2.47 Å), and ARG-180 (2.45 Å, 2.54 Å). Similarly, the hydrogen bonds formed between NCTD and caspase-9 occurred between the SER-287 (2.59 Å, 2.37 Å). These hydrogen bond interactions may provide the fundamental conditions for the reaction of caspase-9 with NCTD and 5-FU. Furthermore, we performed molecular dynamics simulations of both complexes for up to 10 ns [37], which revealed that both small molecules can form hydrogen bonds with caspase-9 (Figure S1D). Hydrogen bonding is a very strong non-covalent interaction that can reflect, to some extent, the binding strength between the ligand and the receptor.

### 3.5. NCTD Enhanced 5-FU-Induced Apoptosis by the Caspase-Dependent Pathway

**Figure 5:**
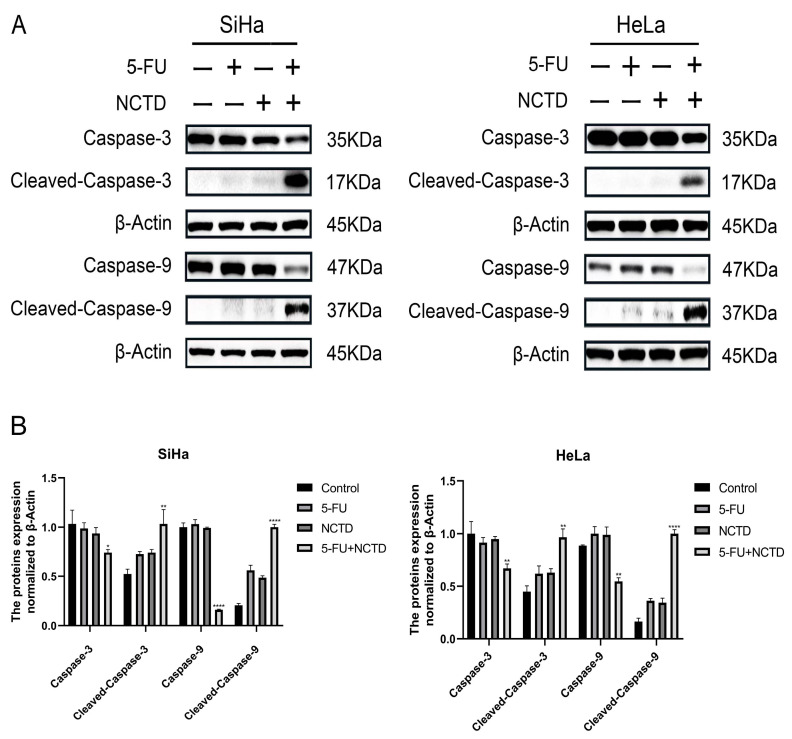
(**A**) Cell lysates were analyzed with caspase-3 and caspase-9 antibodies by immunoblotting analysis. (**B**) The quantified statistical results. GAPDH was used as an internal control for total protein loading. All experiments were performed in triplicate. Date are shown as the mean ± SEM (n = 3): * *p* < 0.05, ** *p* < 0.01, and **** *p* < 0.0001 versus 5-FU group.

Based on network pharmacology, molecular docking, and molecular dynamics simulations, we speculate that the combined effect of NCTD and 5-FU may be achieved by jointly targeting caspase-9. Immunoblotting was performed to examine the apoptosis-related protein caspase-9 in HeLa and SiHa cells. The expression level of cleaved caspase-9 was significantly increased in the NCTD + 5-FU group compared to that in the NCTD or 5-FU alone groups. In addition, their combination caused a greater cleavage of caspase-3 than the drug alone, indicating further enhanced apoptosis (Figure 5A,B). These results suggest that NCTD combined with 5-FU-induced cell apoptosis involves an intrinsic apoptotic pathway.

## 4. Discussion

Some natural products can overcome the shortcomings of chemotherapeutic drugs, such as non-target specificity, undesirable side effects, and high cost. In recent years, the focus on developing anticancer strategies with high efficacy and low toxicity has shifted to a combination of chemotherapeutic agents and natural products. As an effective antitumor drug, NCTD has been used for many years to treat patients with cancers such as liver cancer, breast cancer, colorectal cancer, and leukemia in China [14]. Clinical studies have shown that whether NCTD is administered orally or by intravenous infusion, it can effectively inhibit tumor growth. Moreover, the combination of NCTD with chemotherapeutic drugs such as oxaliplatin, cisplatin, doxorubicin, carboplatin, and other chemotherapeutic drugs not only improves the therapeutic effect of a wide range of cancers but also reduces the occurrence of side effects such as leukopenia liver damage and neutropenia in the clinic and enhances the immune response of patients [14,38], thereby improving the quality of life of patients and prolonging the survival time of patients. Thus, the combination therapy may be an effective method to enhance the therapeutic effect and reduce the side effects of 5-FU.

Data from this study showed that NCTD and 5-FU inhibited the proliferation of HeLa and SiHa cells in a dose- and time-dependent manner and that NCTD combined with 5-FU had a more potent inhibitory effect. Furthermore, the combination treatment resulted in a higher percentage of apoptosis than either NCTD or 5-FU alone. Currently, comprehensive analyses using bioinformatics methods including network pharmacology, molecular docking, and molecular dynamics simulations have become an effective tool for exploring the potential mechanisms of natural products, which play a key role in drug discovery. For example, network pharmacology analysis was used to screen key pharmacological targets, followed by molecular docking to predict the binding affinity of the drug to the screened protein targets, which can be used to initially verify the accuracy of the predicted targets. Then, molecular dynamics simulations were used to evaluate the accurate binding mode and binding ability of the docked conformations, which were then verified experimentally so that the rational use of bioinformatics could help to accelerate the drug discovery process. In this study, based on the collection of apoptosis genes in the GeneCards database and the identification of drug targets for NCTD and 5-FU by TargetNet prediction software, the three gene clusters were cross-analyzed to obtain a Venn diagram (Figure 4A), which showed the intersections of the drugs with 19 targets of apoptosis, and a PPI network was constructed (Figure 4B). The top ten key targets calculated by Cytoscape software were PTGS1, CES1, NOS3, SIRT2, AHR, CYP19A1, CYP1A2, CA12, CASP9, and BCL2A1, respectively. caspase-9 is a key caspase in the intrinsic apoptosis pathway. Molecular docking analysis showed that both NCTD and 5-FU have a strong binding affinity for caspase-9, respectively. But, this should be further investigated through competitive binding assays. Subsequently, in a molecular dynamics run at 10 ns, the complex system of both drugs with caspase-9 behaved as stable, and both formed hydrogen bonds with caspase-9 (Figure S1D). Moreover, the RMSD can indicate the conformation change and the system stability of the complexes, and the Rg value can indicate the tightness of the complex system, which can reflect the degree of protein folding. The results of RMSD and Rg analyses showed that the caspase-9-5-FU complex and caspase-9-NCTD complex reached dynamic equilibrium, and both had small fluctuations, indicating that the small molecules were able to bind stably to the target proteins (Figure S1A,C). Also, the RMSF can be used to indicate fluctuations in the complexes at the residue level. The results showed that most amino acid residues of both complexes had RMSF values of less than 5 Å (Figure S1B). Most of the amino acid conformational changes were small during the simulation, and only a few residues experienced large conformational changes, most likely because they were located in the hinge region of the protein. The RMSF values of the two complexes showed almost the same peaks and valleys, suggesting that the two small molecules acted similarly on the target receptor and they may have similar pharmacodynamic mechanisms. Also, NCTD and 5-FU also showed a strong binding affinity for CA12 and PTGS1 (Table S1), which will be explored in our future studies.

Previous studies have shown that certain factors or conditions can activate caspase-9 [14]. Activated caspase-9 cleaves and activates effector caspase-3, where the activation of these enzymes triggers a cascade of reactions that leading to the degradation of cellular components and ultimately to cell death [39]. This process is essential for maintaining tissue homeostasis and eliminating damaged or infected cells. However, the inappropriate activation of these enzymes can also lead to pathological conditions such as neurodegeneration and cancer. Therefore, understanding the mechanisms that regulate caspase activation is crucial for developing effective therapeutic strategies against these diseases [40,41].

However, these preliminary results require further validation. The safety and feasibility of the drug combinations need to be further investigated in clinical studies to assess drug response in the short term and potential toxic side effects in the long term. In addition, it would be interesting to further investigate the mechanism of synergistic effects.

## 5. Conclusions

In conclusion, we demonstrated that NCTD acts synergistically with 5-FU to inhibit the proliferation of cervical cancer cells. NCTD enhances the apoptotic effect of 5-FU on cervical cancer cells via a caspase-dependent pathway, which is consistent with the predictions of network pharmacology and molecular docking. In addition, as 5-FU and NCTD have been used clinically, our findings may provide an alternative regimen for the treatment of cervical cancer.

## Data Availability

Data are contained within the article.

## Supplementary Materials

The following supporting information can be downloaded at: https://www.mdpi.com/article/10.3390/cimb46050242/s1. Table S1. Molecular docking results of 8 key expressed proteins. Figure S1. Molecular Dynamics Simulation results of caspase-9-5-FU complex and caspase-9-NCTD complex, respectively. (A) Root Mean Square Deviation. (B) Root Mean Square Fluctuation. (C) Radius of Gyration. (D) The number of hydrogen bonds.
