# Supplementary material for: Norcantharidin Enhances the Antitumor Effect of 5-Fluorouracil by Inducing Apoptosis of Cervical Cancer Cells: Network Pharmacology, Molecular Docking, and Experimental Validation
Source: Curr Issues Mol Biol. 2024 Apr 25;46(5):3906–18. doi: 10.3390/cimb46050242 (PMC11120450; doi:10.3390/cimb46050242)
Supplement: Supplementary file 1 [file cimb-46-00242-s001.zip › cimb-2949536-supplementary.pdf]

GENE	Uniprot ID	PDB ID	ΔG (kcal/mol)	
			5-Fluorouracil	norcantharidin
BCL2A1	Q16548	6vo4	-4.1	-5.2
CA12	O43570	5ll5	-6.1	-6.9
CASP9	P55211	2ar9	-5.5	-7.1
CES1	P23141	5a7g	-5.2	-6.1
CYP19A1	P11511	3s79	-5.1	-5.4
CYP1A2	P05177	2hi4	-5.7	-6.2
PTGS1	P23219	6y3c	-6.2	-6.8
SIRT2	Q8IXJ6	5y5n	-5.0	-5.5

Table S1. Molecular docking results of 8 key expressed proteins.

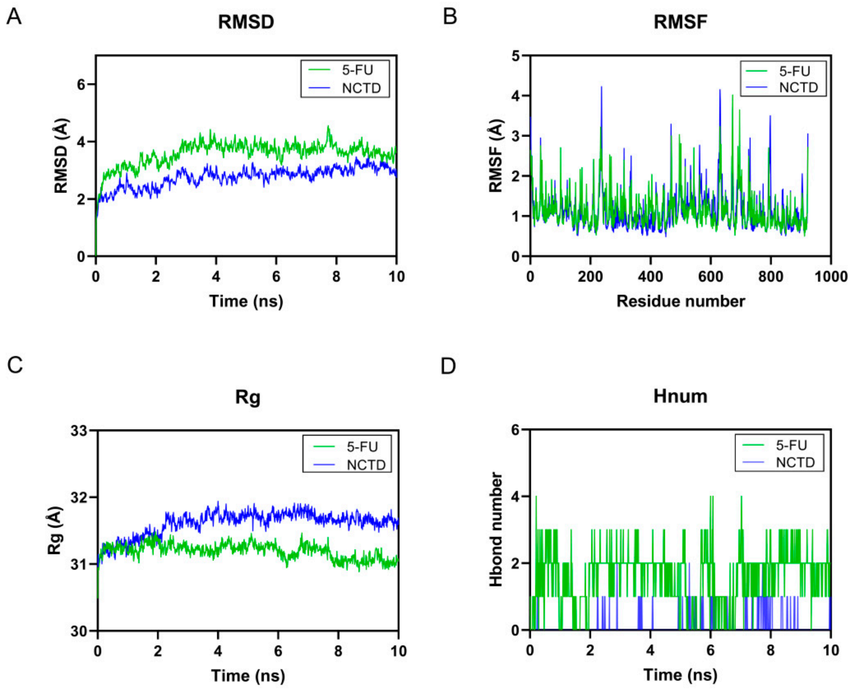

Figure S1. Molecular Dynamics Simulation results of caspase-9-5-FU complex and caspase-9-NCTD complex, respectively. (A) Root Mean Square Deviation. (B) Root Mean Square Fluctuation. (C) Radius of Gyration. (D)The number of hydrogen bonds.
